# Supplementary material for: Syntheses of New Multisubstituted 1-Acyloxyindole Compounds
Source: Molecules. 2022 Oct 10;27(19):6769. doi: 10.3390/molecules27196769 (PMC9570991; doi:10.3390/molecules27196769)
Supplement: Supplementary file 1 [file molecules-27-06769-s001.zip › molecules-1957779-supplementary.pdf]

Supplementary Materials

for

Syntheses of new multisubstituted 1-acyloxyindole compounds

Ye Eun Kim, Yoo Jin Lim, Chorong Kim, Yu Ra Jeong, Hyunsung Cho and Sang Hyup Lee*

College of Pharmacy and Innovative Drug Center, Duksung Women's University, Seoul 01369, Republic of Korea,

*sanghyup@duksung.ac.kr

^{1}H and ^{13}C NMR spectrum of compound **1ax** ------------------------------------ S3

^{1}H and ^{13}C NMR spectrum of compound **1ay** ------------------------------------ S4

^{1}H and ^{13}C NMR spectrum of compound **1az** ------------------------------------ S5

^{1}H and ^{13}C NMR spectrum of compound **1bx** ------------------------------------ S6

^{1}H and ^{13}C NMR spectrum of compound **1by** ------------------------------------ S7

^{1}H and ^{13}C NMR spectrum of compound **1bz** ------------------------------------ S8

^{1}H and ^{13}C NMR spectrum of compound **1cx** ------------------------------------ S9

^{1}H and ^{13}C NMR spectrum of compound **1cy** ----------------------------------- S10

^{1}H and ^{13}C NMR spectrum of compound **1cz** ----------------------------------- S11

^{1}H and ^{13}C NMR spectrum of compound **1du** ---------------------------------- S12

^{1}H and ^{13}C NMR spectrum of compound **1dv** ---------------------------------- S13

^{1}H and ^{13}C NMR spectrum of compound **1dw** --------------------------------- S14

^{1}H and ^{13}C NMR spectrum of compound **1dx** ---------------------------------- S15

^{1}H and ^{13}C NMR spectrum of compound **1dy** --------------------------------- S16

^{1}H and ^{13}C NMR spectrum of compound **1dz** --------------------------------- S17

^{1}H and ^{13}C NMR spectrum of compound **1ex** --------------------------------- S18

^{1}H and ^{13}C NMR spectrum of compound **1ey** --------------------------------- S19

^{1}H and ^{13}C NMR spectrum of compound **1ez** --------------------------------- S20

^{1}H and ^{13}C NMR spectrum of compound **1fx** --------------------------------- S21

^{1}H and ^{13}C NMR spectrum of compound **1fy** --------------------------------- S22

^{1}H and ^{13}C NMR spectrum of compound **1fz** --------------------------------- S23

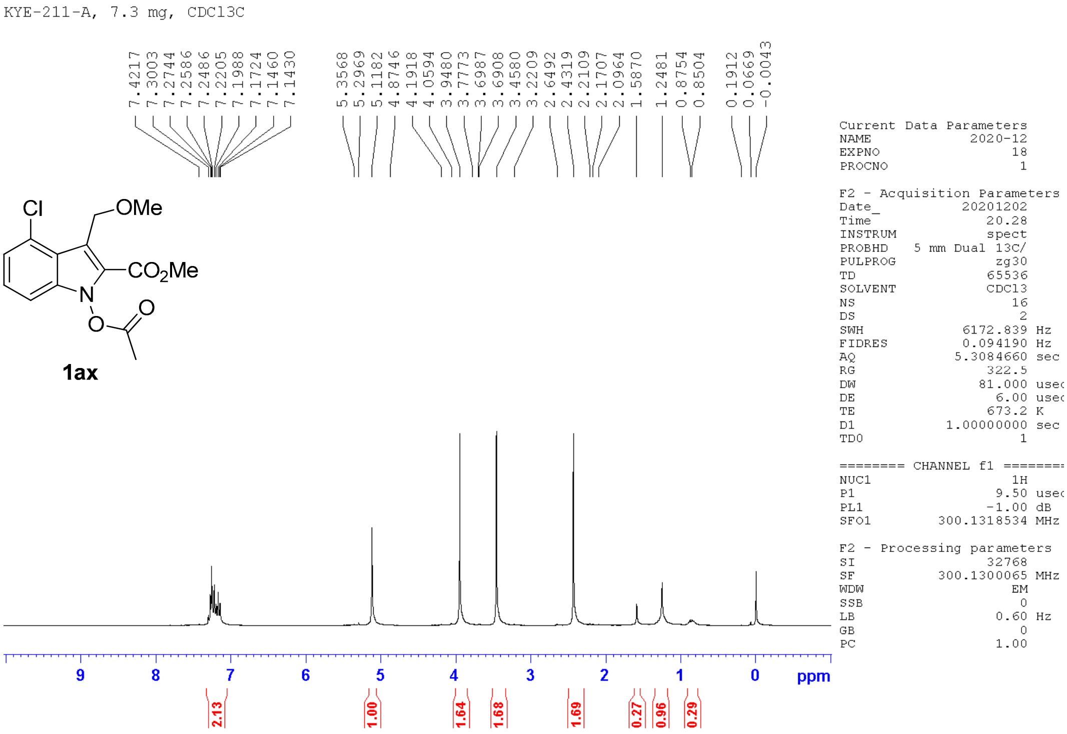

^{1}H NMR spectrum (300 MHz, $CDCl_3$) of compound 1ax

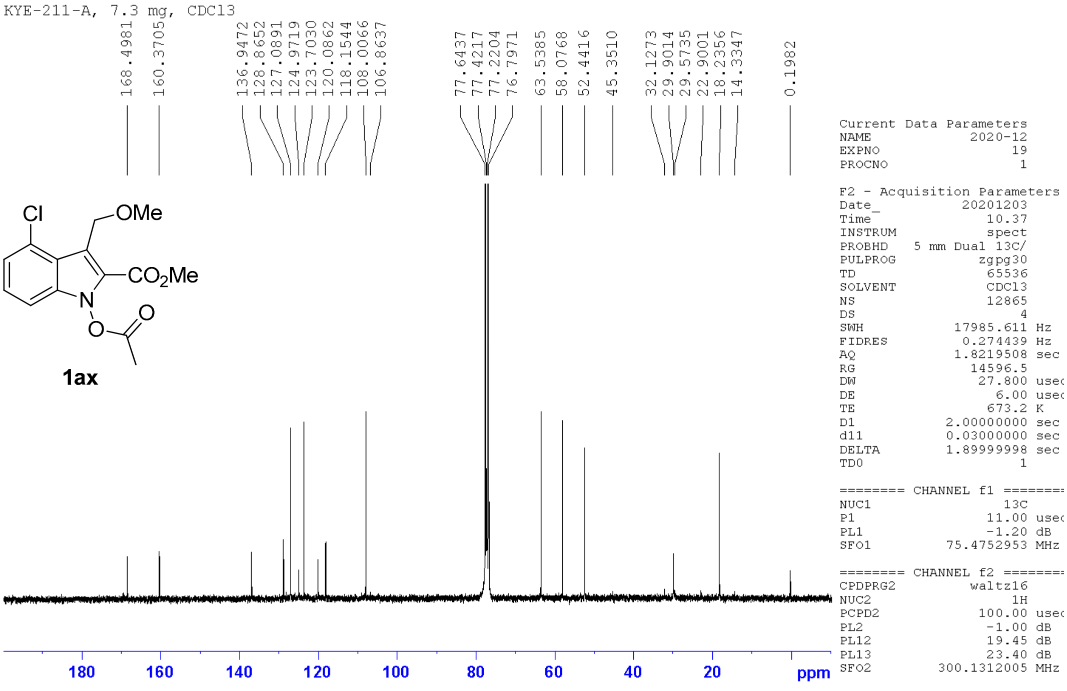

^{13}C NMR spectrum (75 MHz, $CDCl_3$) of compound 1ax

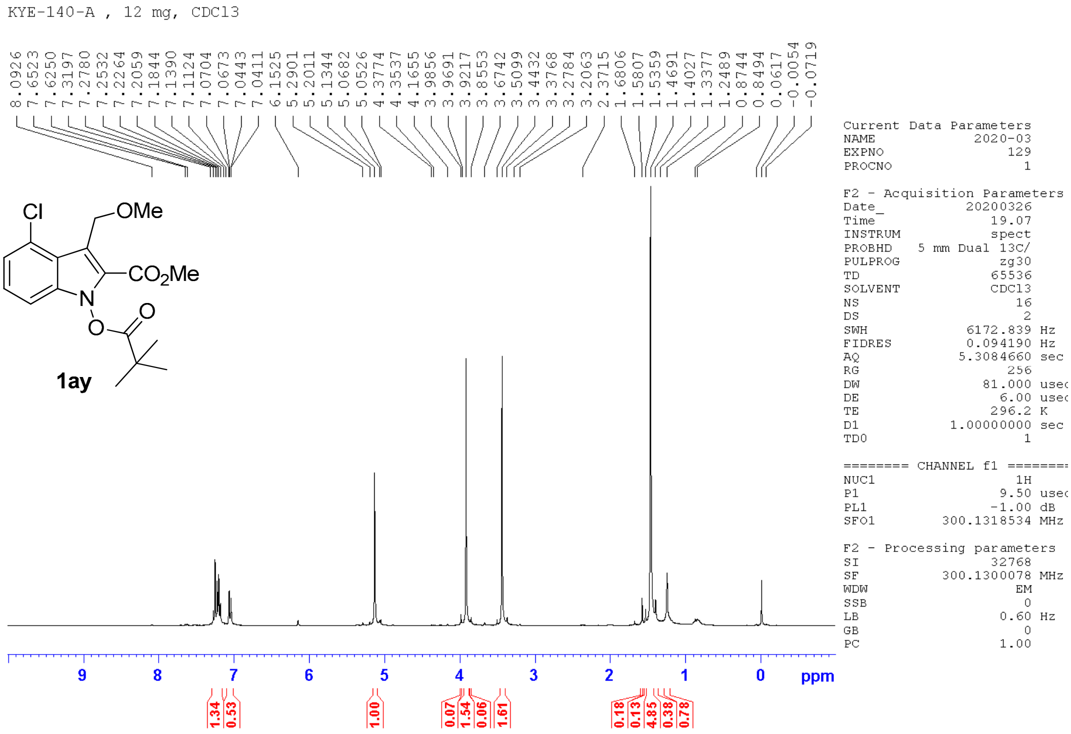

^{1}H NMR spectrum (300 MHz, $CDCl_3$) of compound 1ay

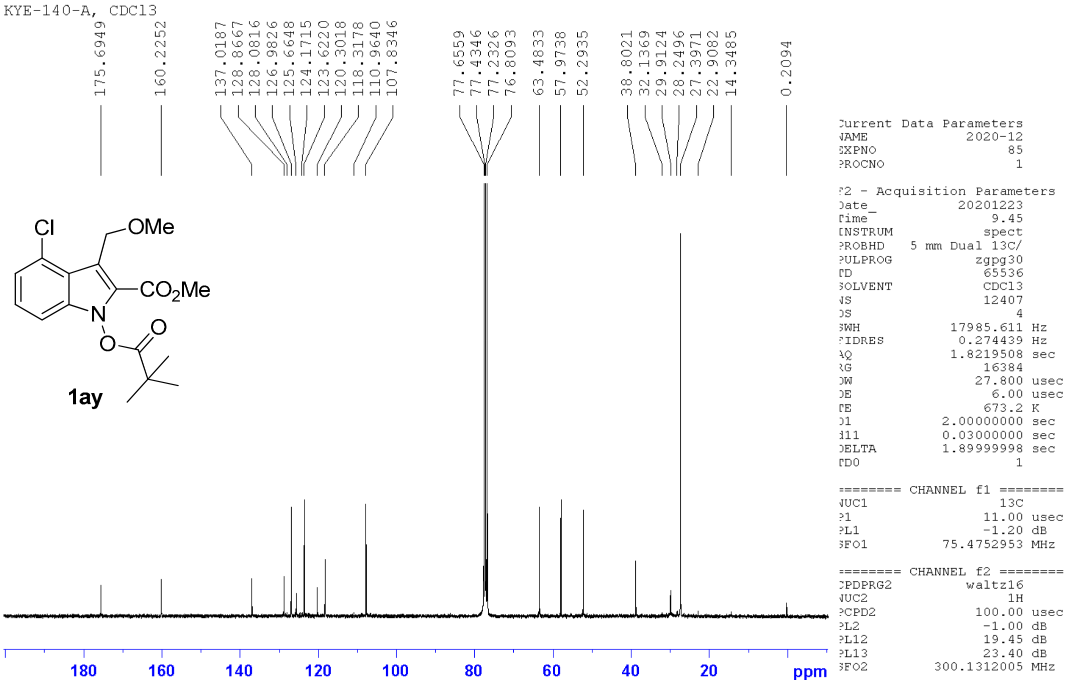

^{13}C NMR spectrum (75 MHz, $CDCl_3$) of compound 1ay

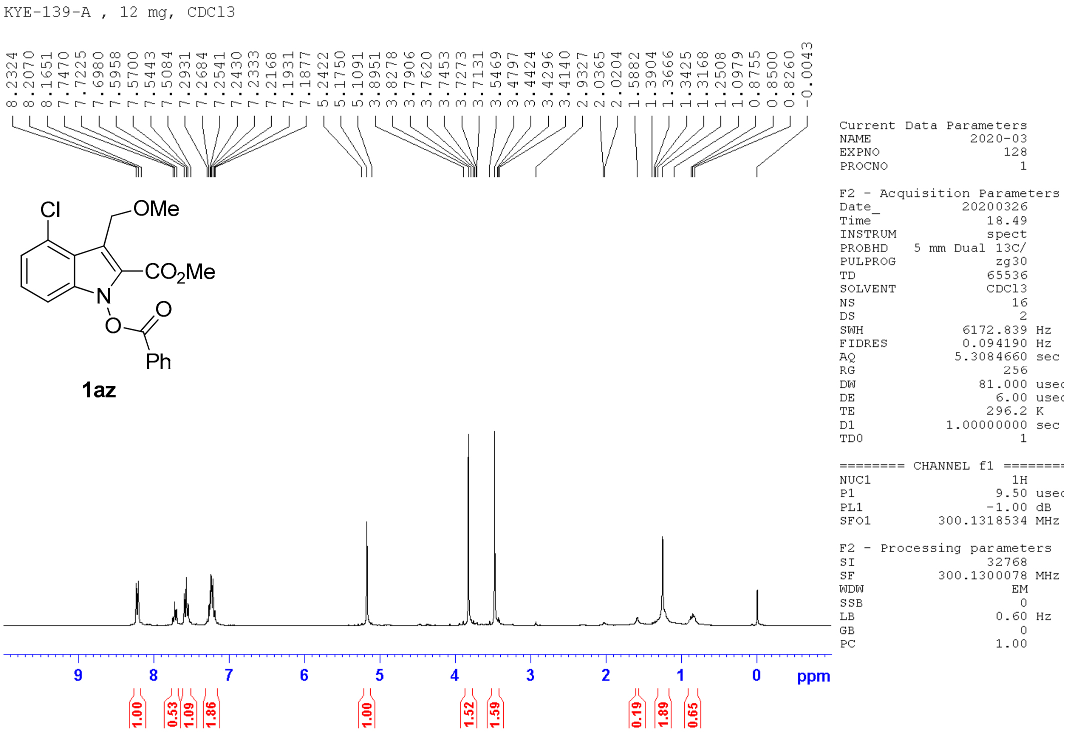

^{1}H NMR spectrum (300 MHz, $CDCl_3$) of compound 1az

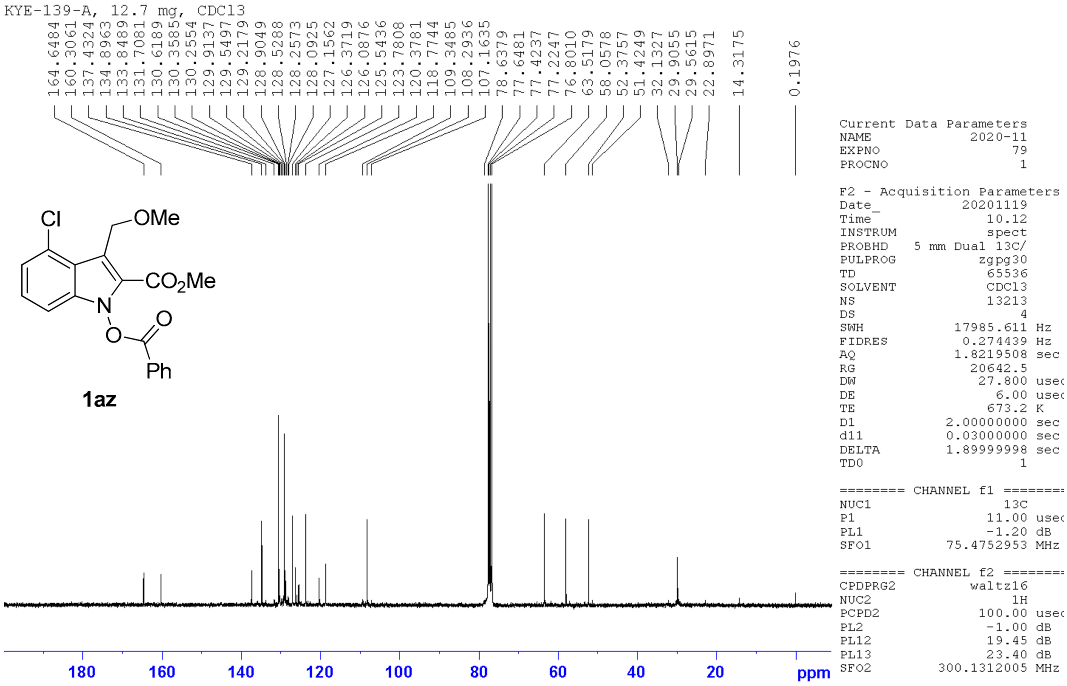

^{13}C NMR spectrum (75 MHz, $CDCl_3$) of compound 1az

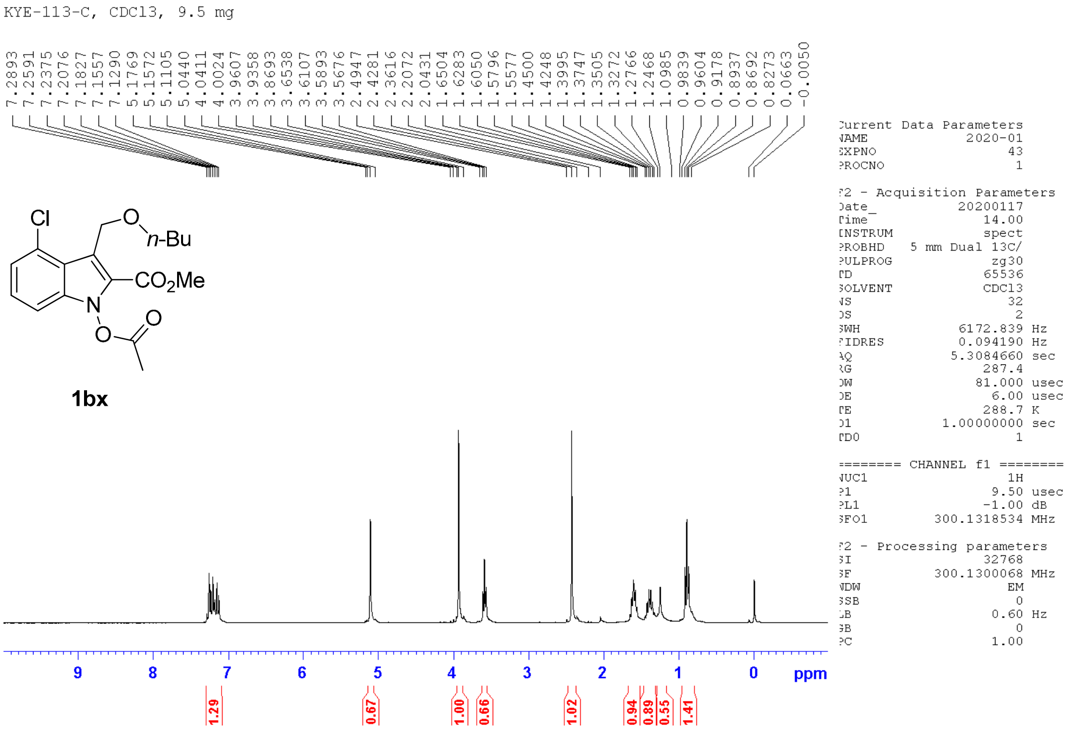

^{1}H NMR spectrum (300 MHz, $CDCl_3$) of compound 1bx

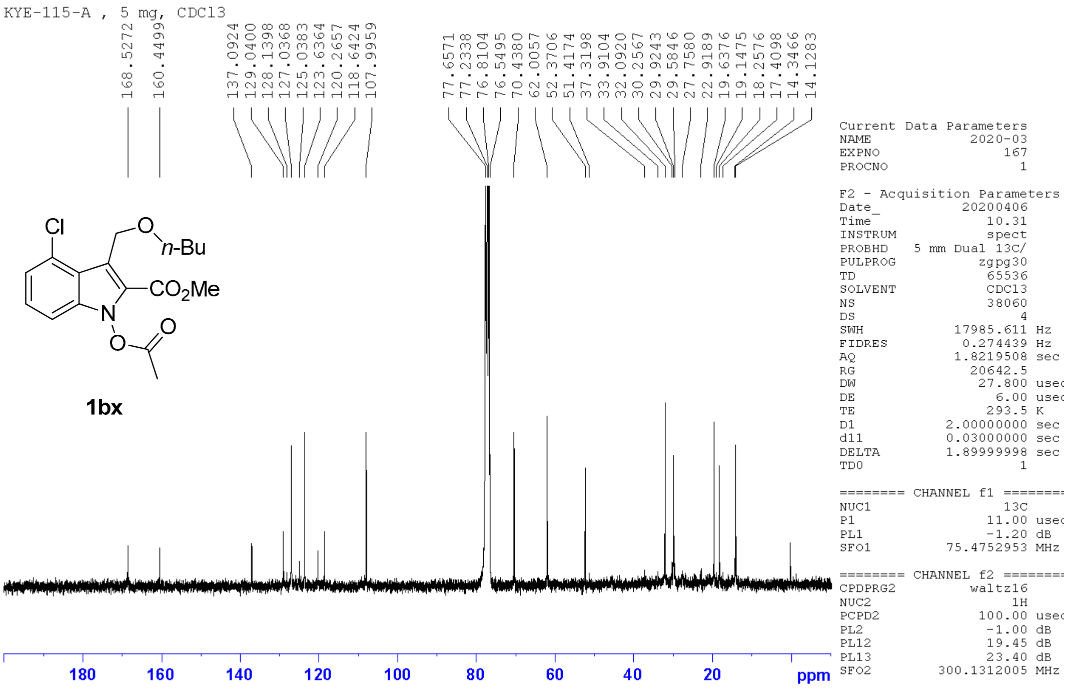

^{13}C NMR spectrum (75 MHz, $CDCl_3$) of compound 1bx

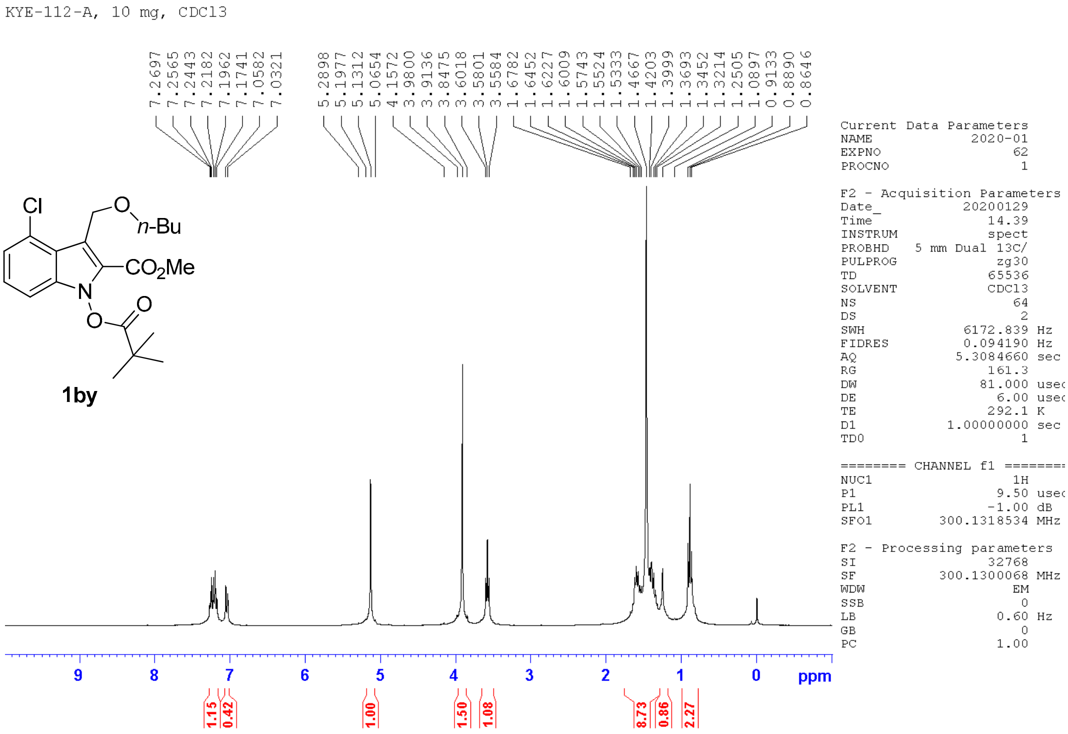

^{1}H NMR spectrum (300 MHz, $CDCl_3$) of compound 1by

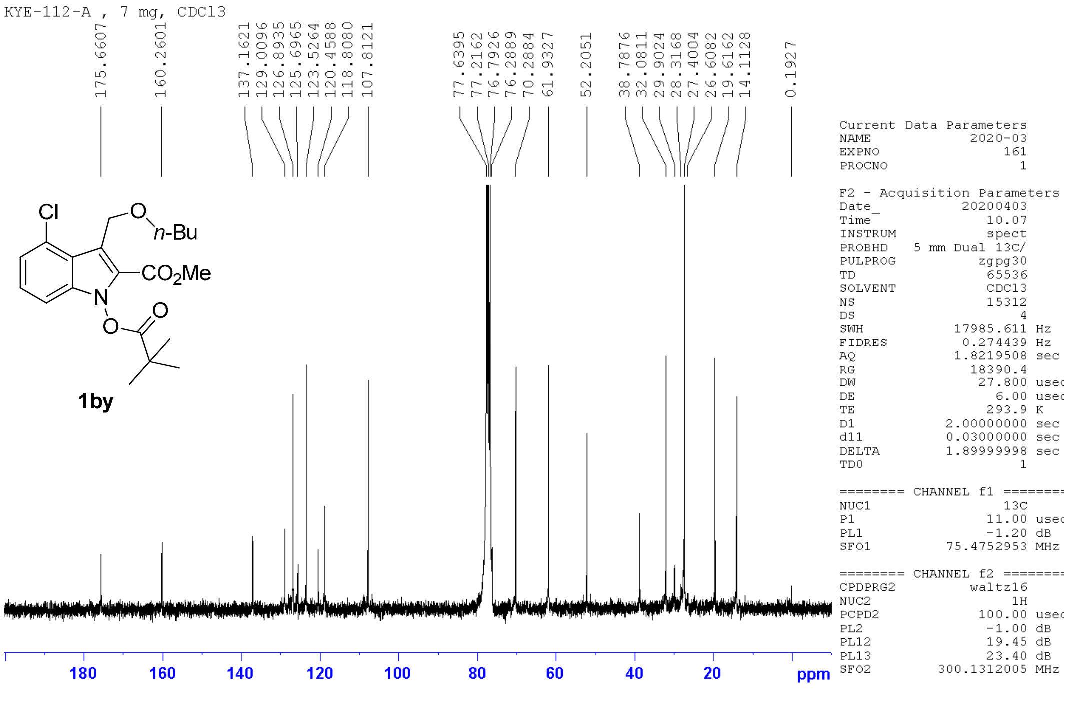

^{13}C NMR spectrum (75 MHz, $CDCl_3$) of compound 1by

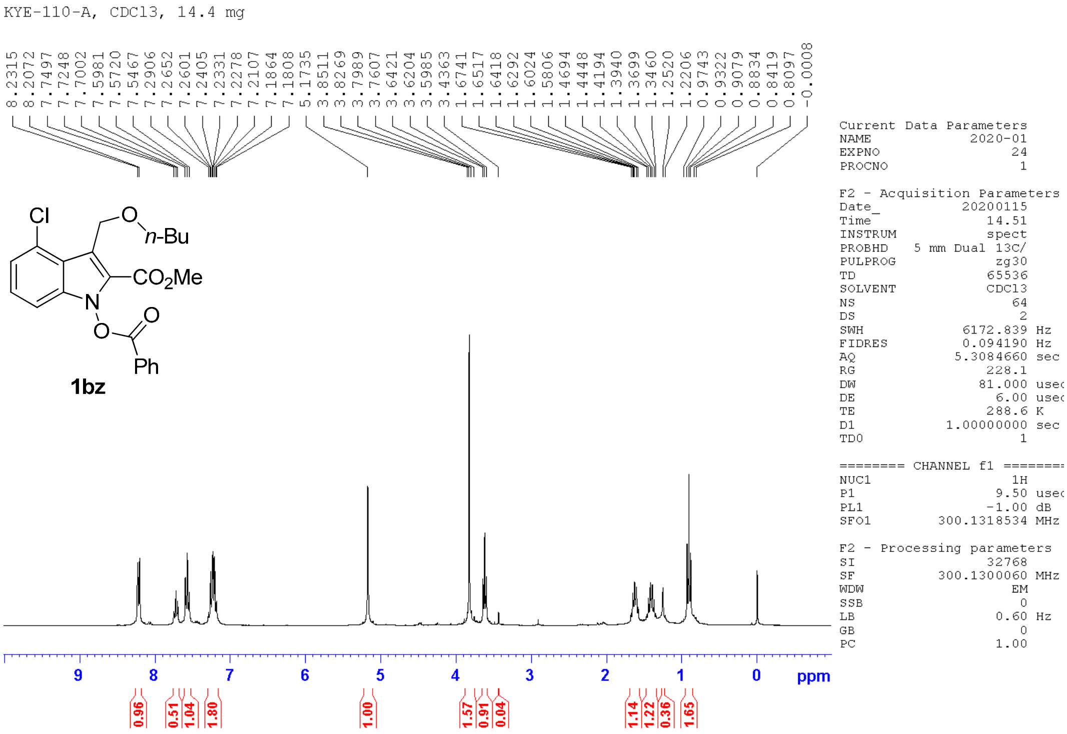

^{1}H NMR spectrum (300 MHz, $CDCl_3$) of compound 1bz

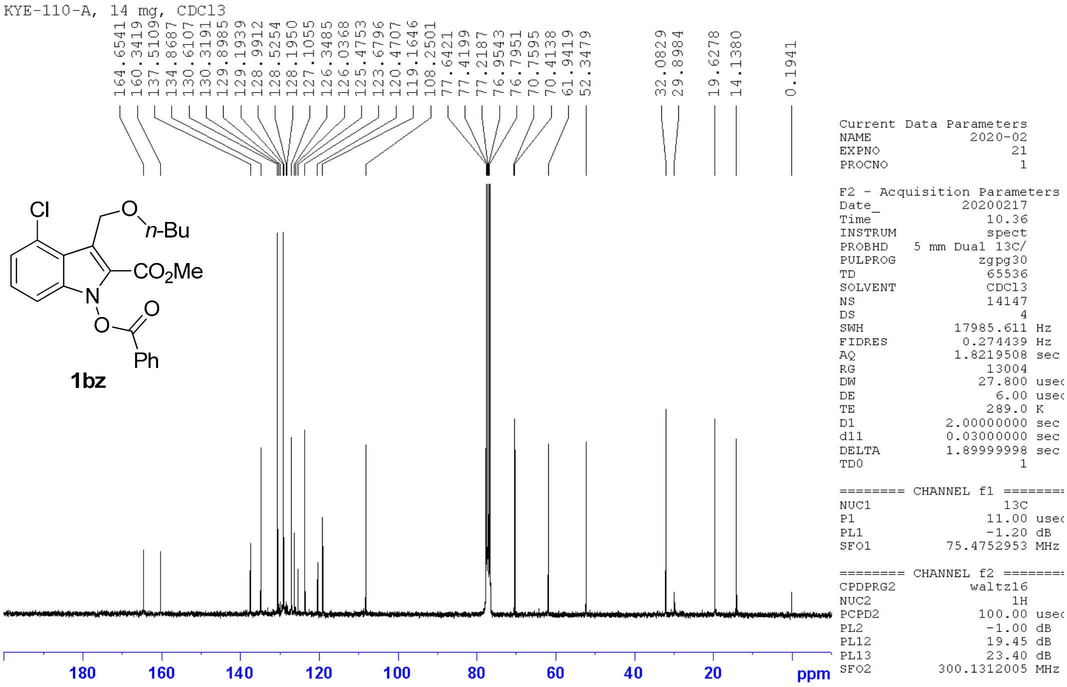

^{13}C NMR spectrum (75 MHz, $CDCl_3$) of compound 1bz

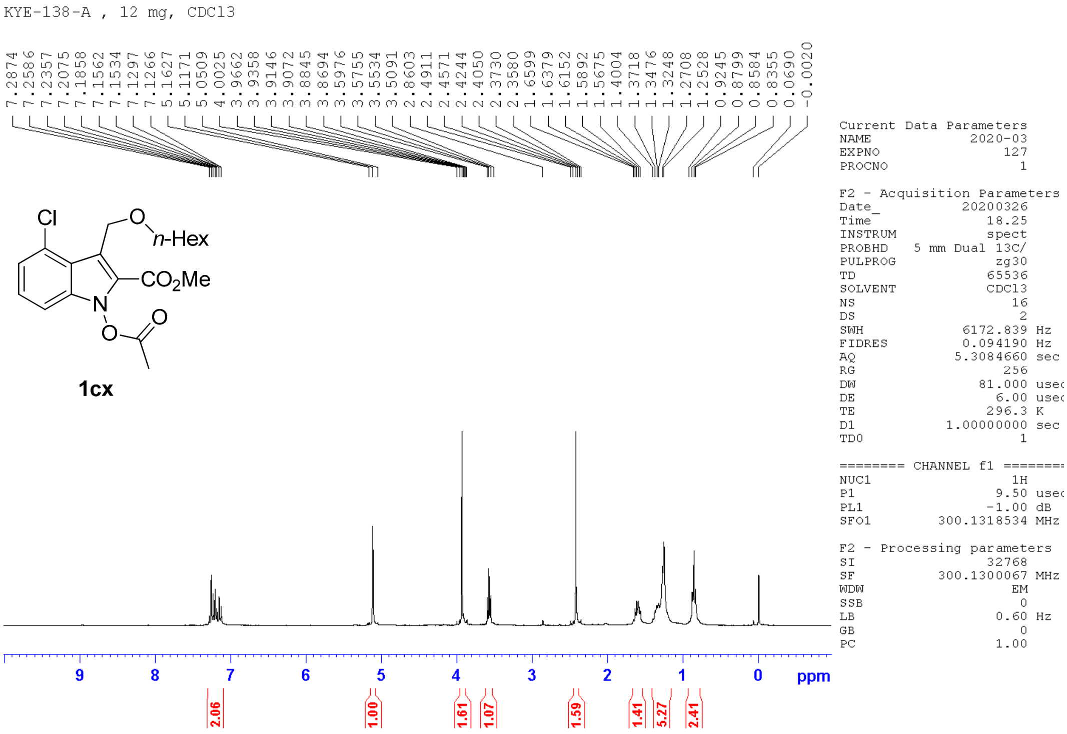

^1H NMR spectrum (300 MHz, $CDCl_3$) of compound 1cx

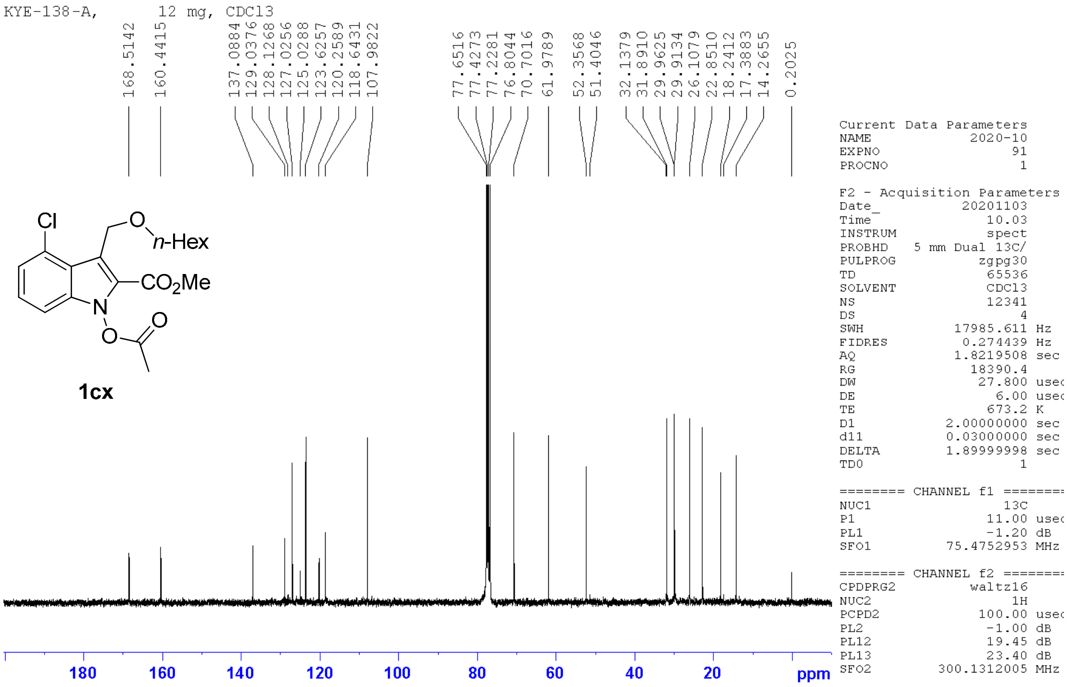

^{13}C NMR spectrum (75 MHz, $CDCl_3$) of compound 1cx

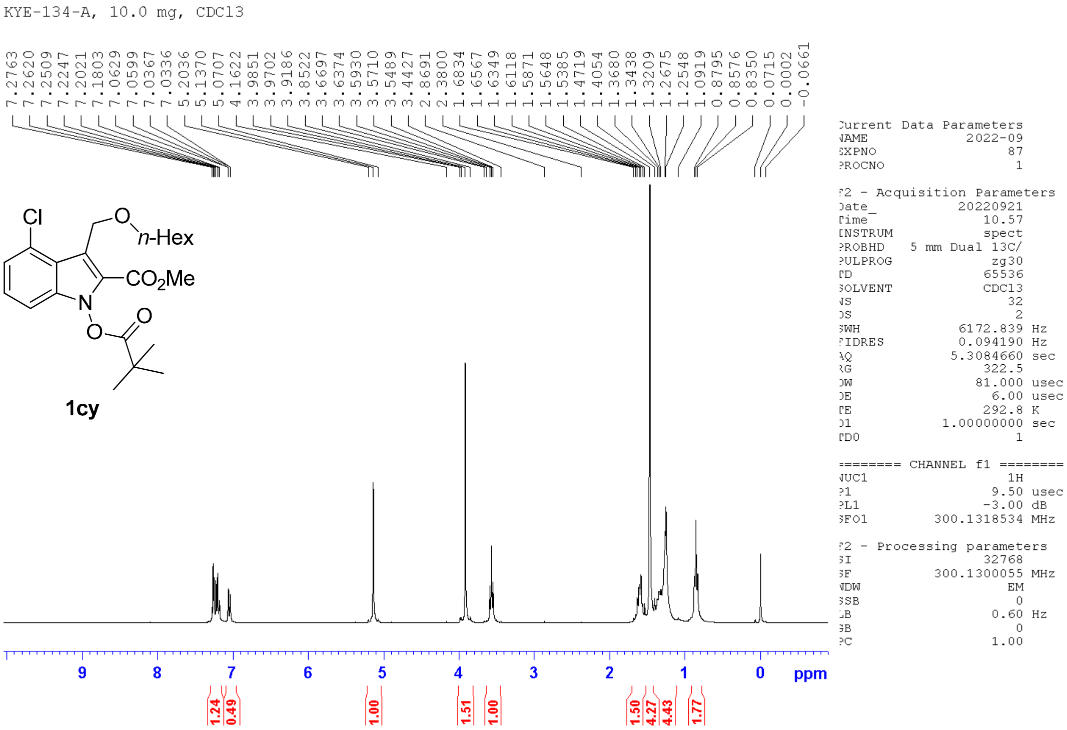

^{1}H NMR spectrum (300 MHz, $CDCl_3$) of compound 1cy

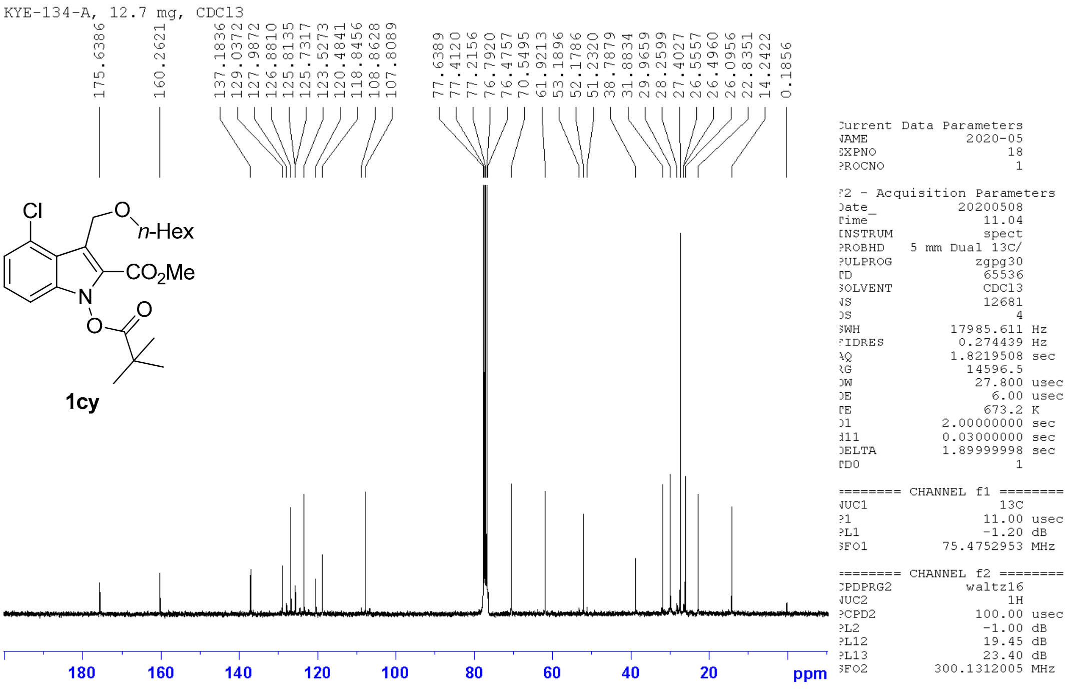

^{13}C NMR spectrum (75 MHz, $CDCl_3$) of compound 1cy

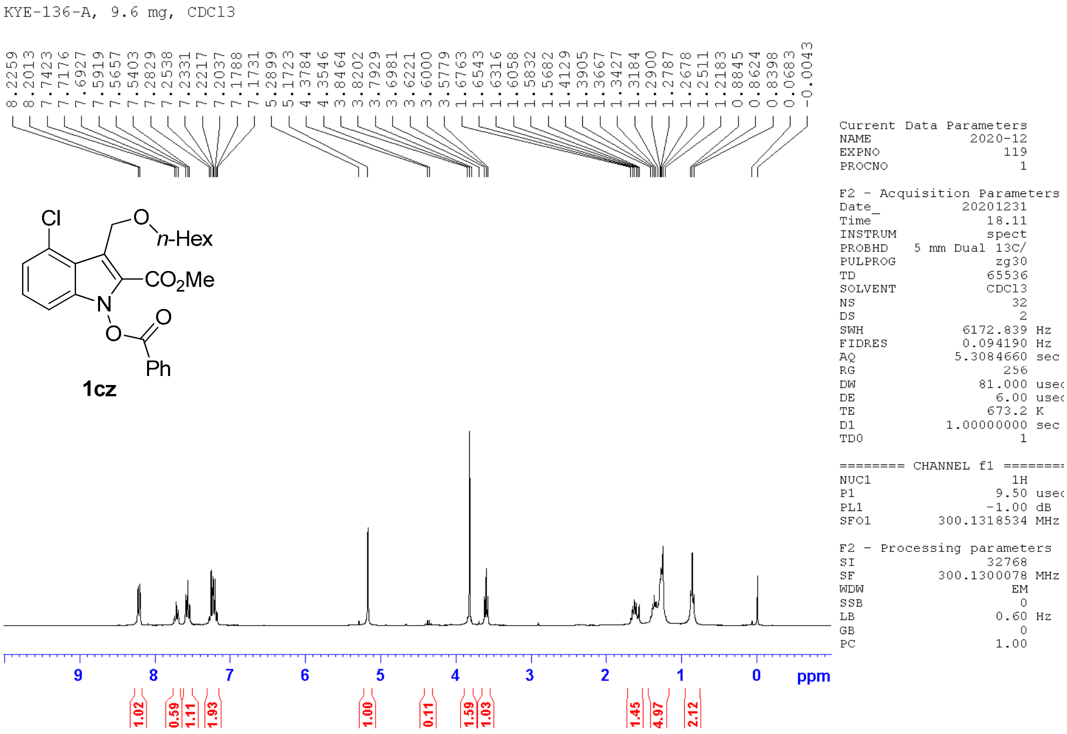

^1H NMR spectrum (300 MHz, $CDCl_3$) of compound 1cz

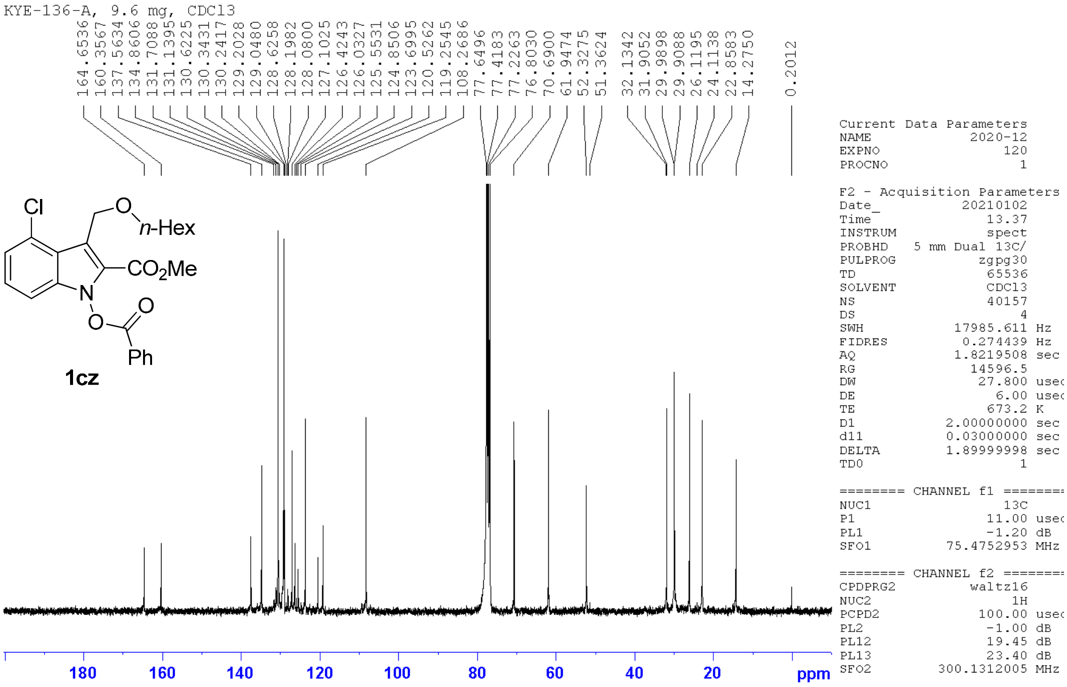

^{13}C NMR spectrum (75 MHz, $CDCl_3$) of compound 1cz

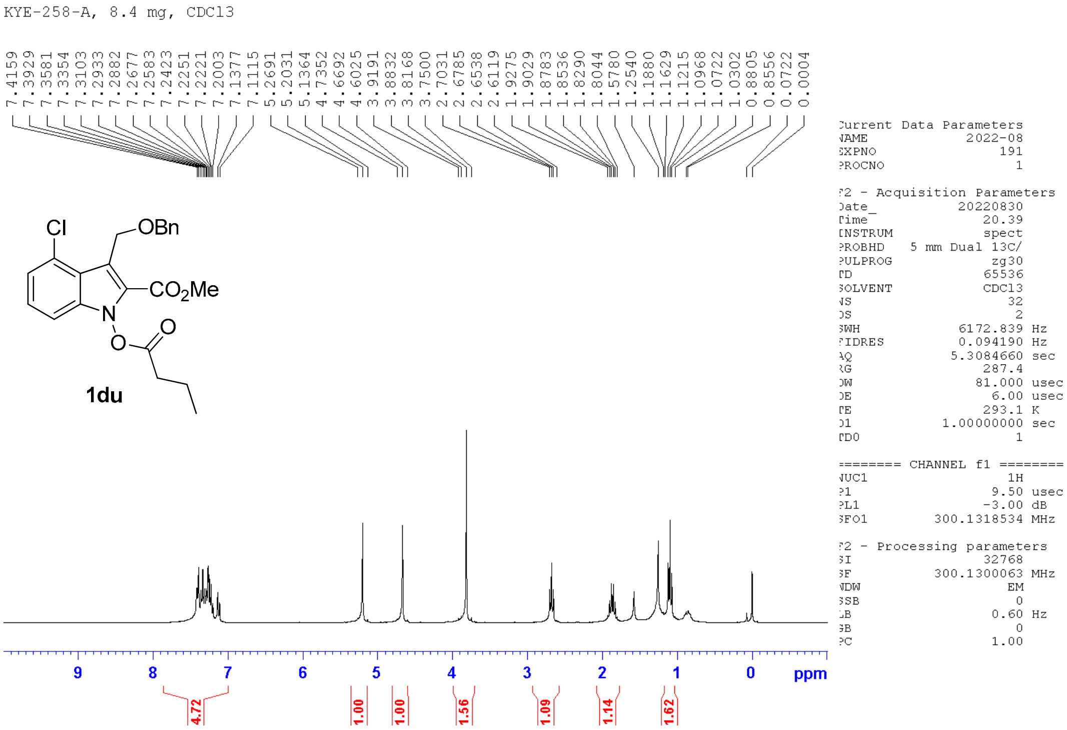

^{1}H NMR spectrum (300 MHz, $CDCl_3$) of compound 1du

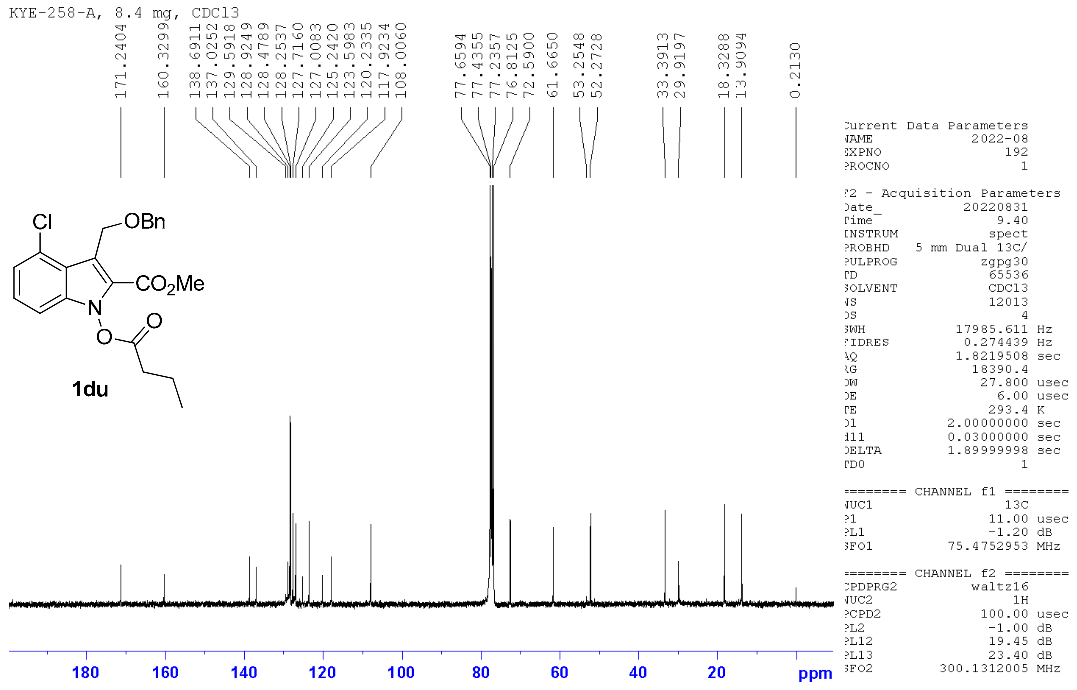

^{13}C NMR spectrum (75 MHz, $CDCl_3$) of compound 1du

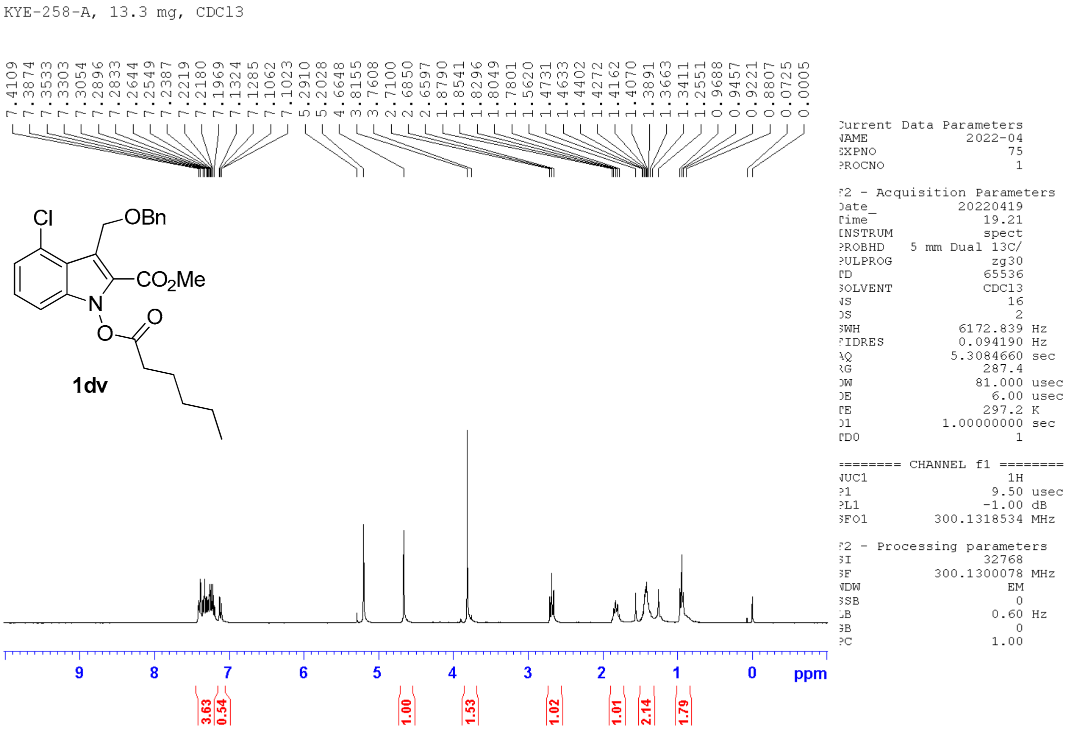

^{1}H NMR spectrum (300 MHz, $CDCl_3$) of compound 1dv

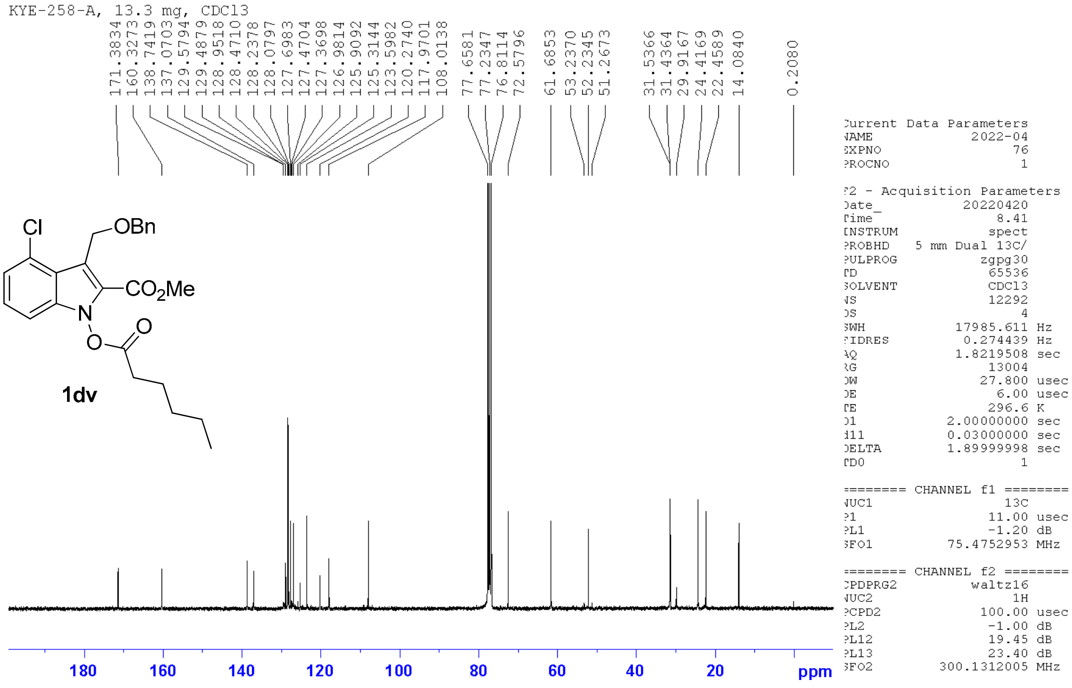

^{13}C NMR spectrum (75 MHz, $CDCl_3$) of compound 1dv

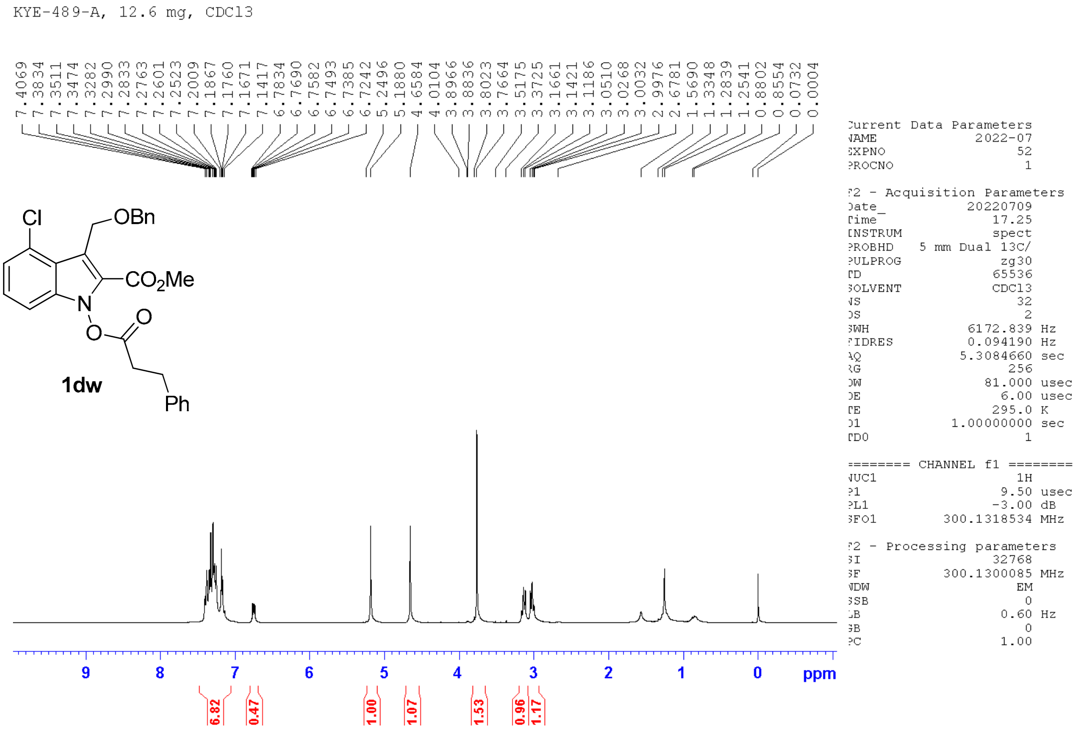

^{1}H NMR spectrum (300 MHz, $CDCl_3$) of compound 1dw

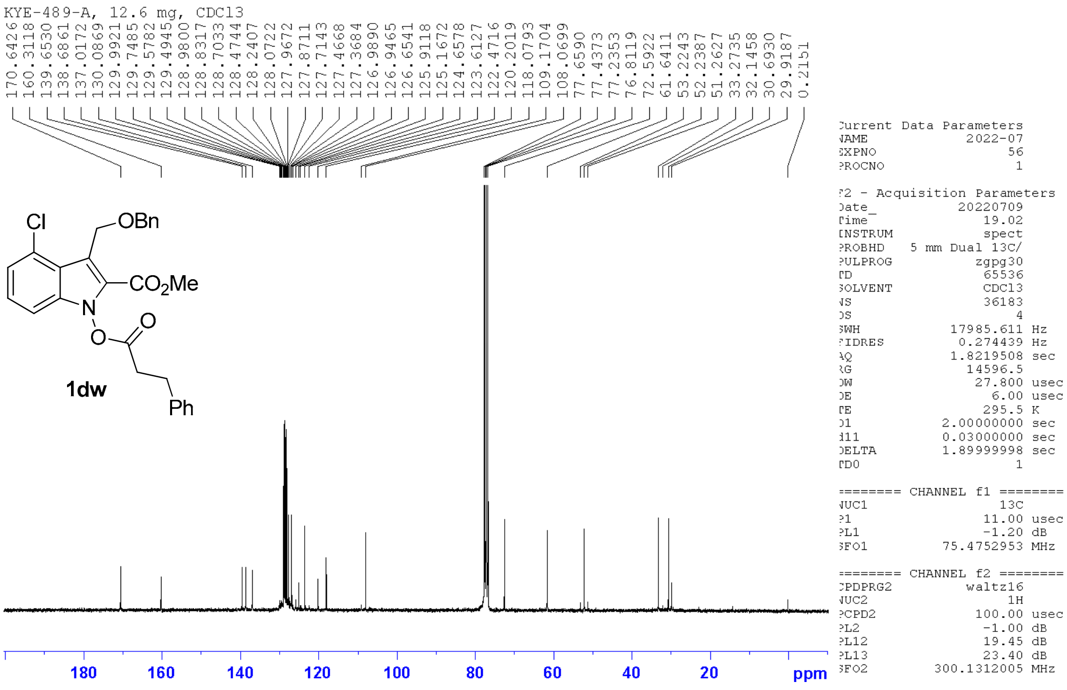

^{13}C NMR spectrum (75 MHz, $CDCl_3$) of compound 1dw

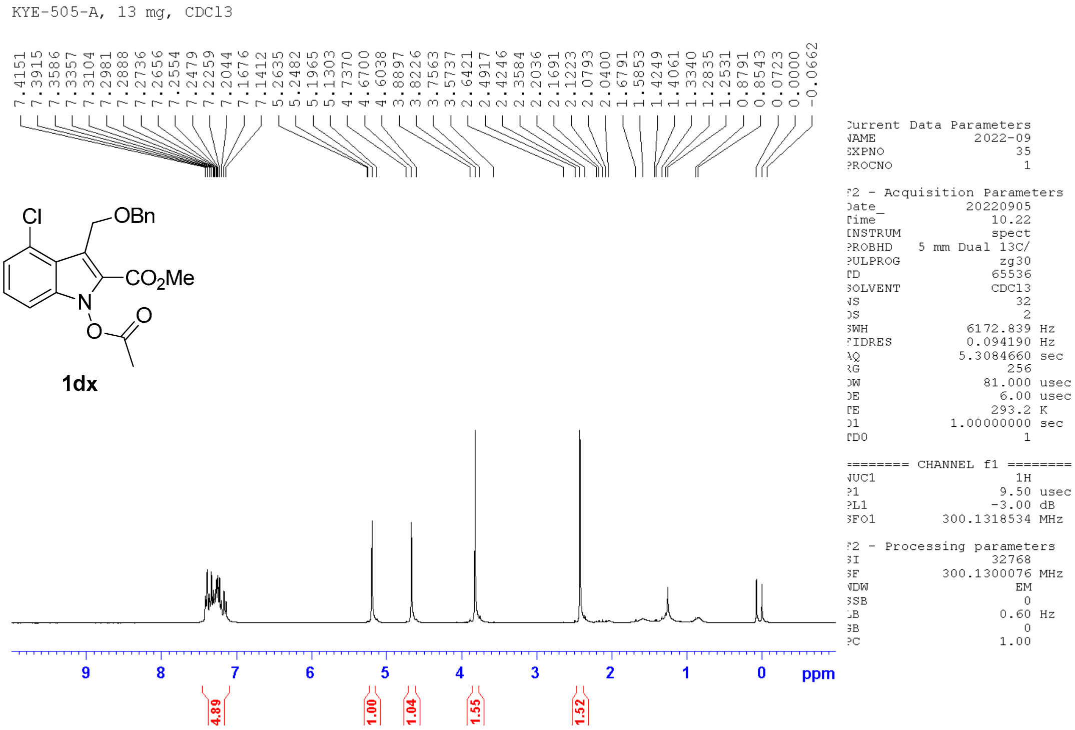

^{1}H NMR spectrum (300 MHz, $CDCl_3$) of compound 1dx

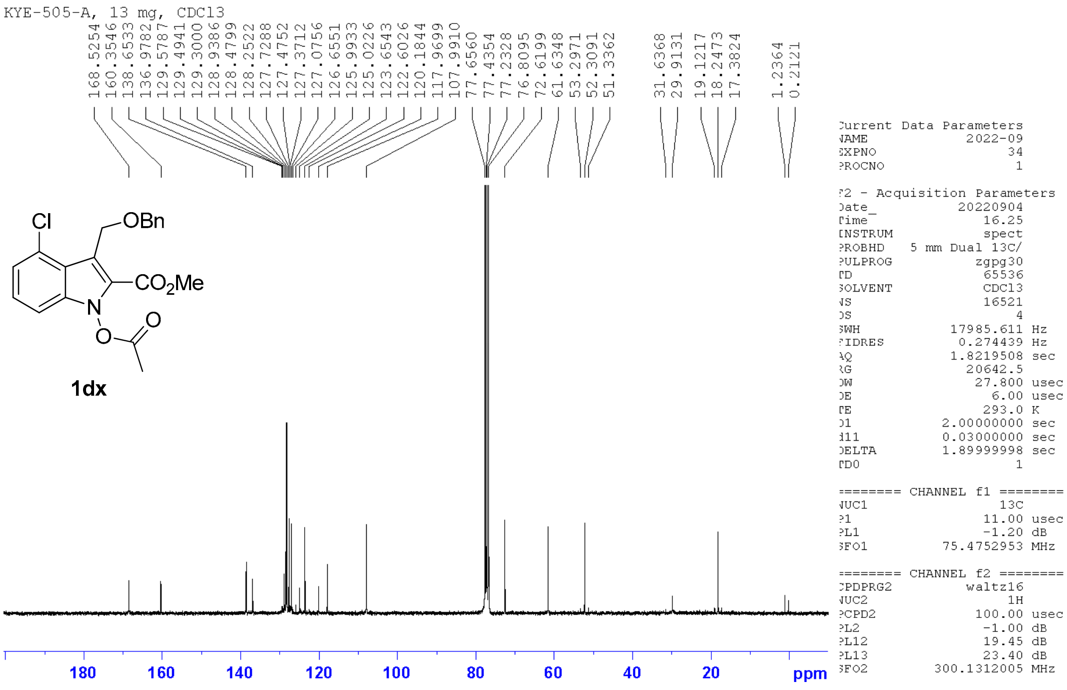

^{13}C NMR spectrum (75 MHz, $CDCl_3$) of compound 1dx

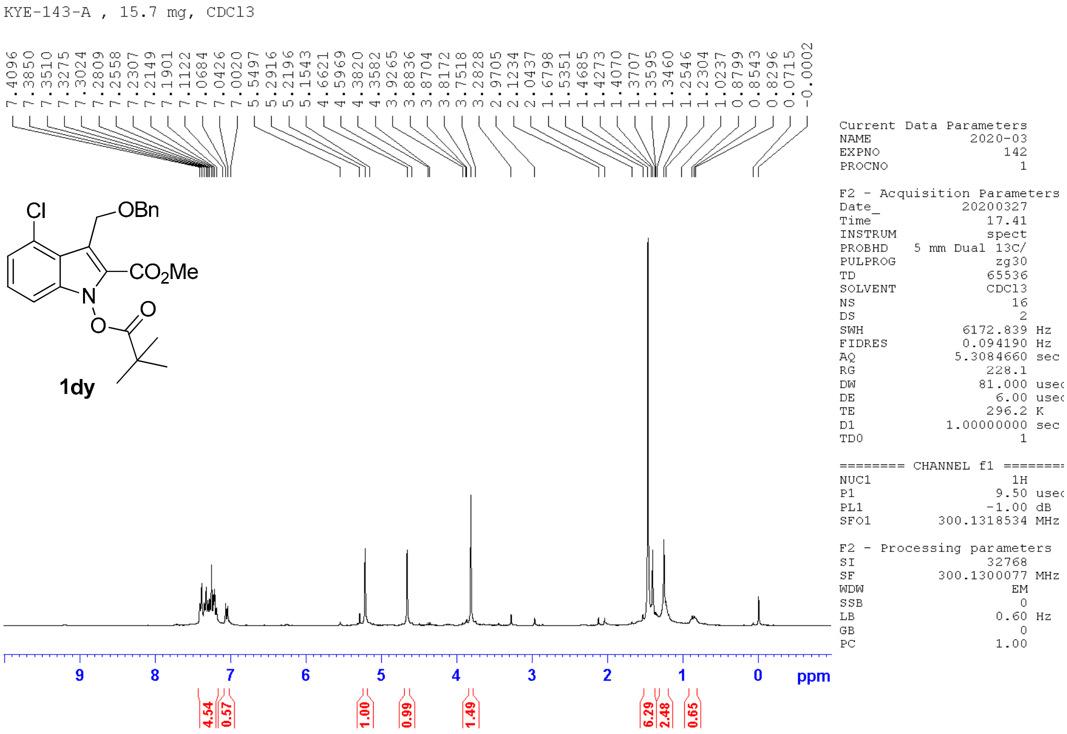

^{1}H NMR spectrum (300 MHz, $CDCl_3$) of compound 1dy

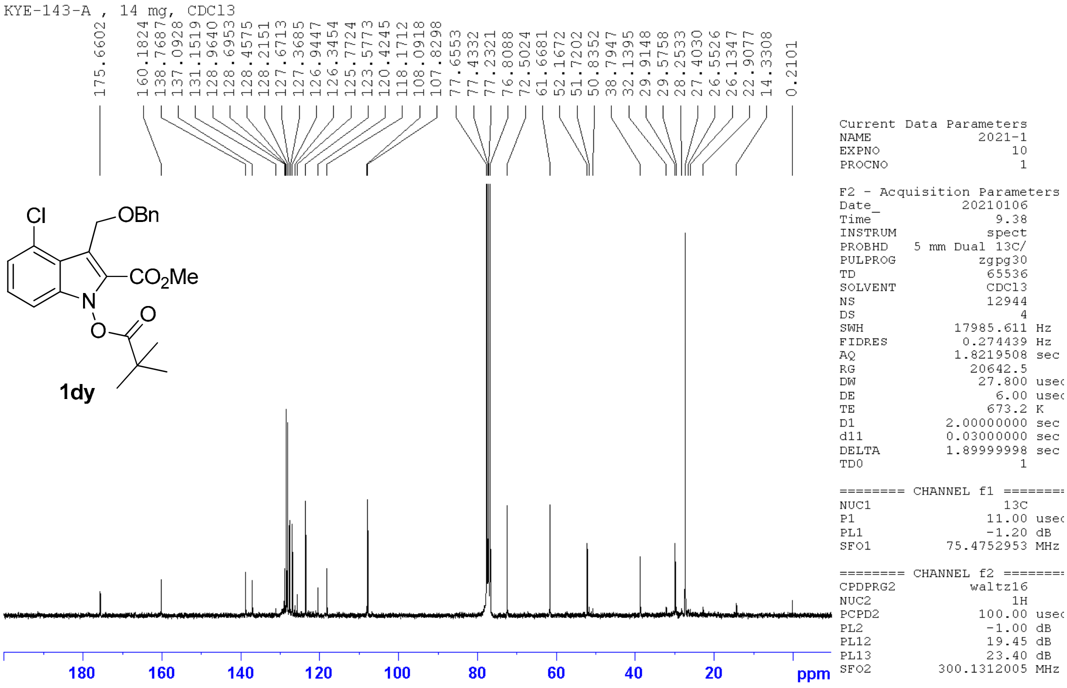

^{13}C NMR spectrum (75 MHz, $CDCl_3$) of compound 1dy

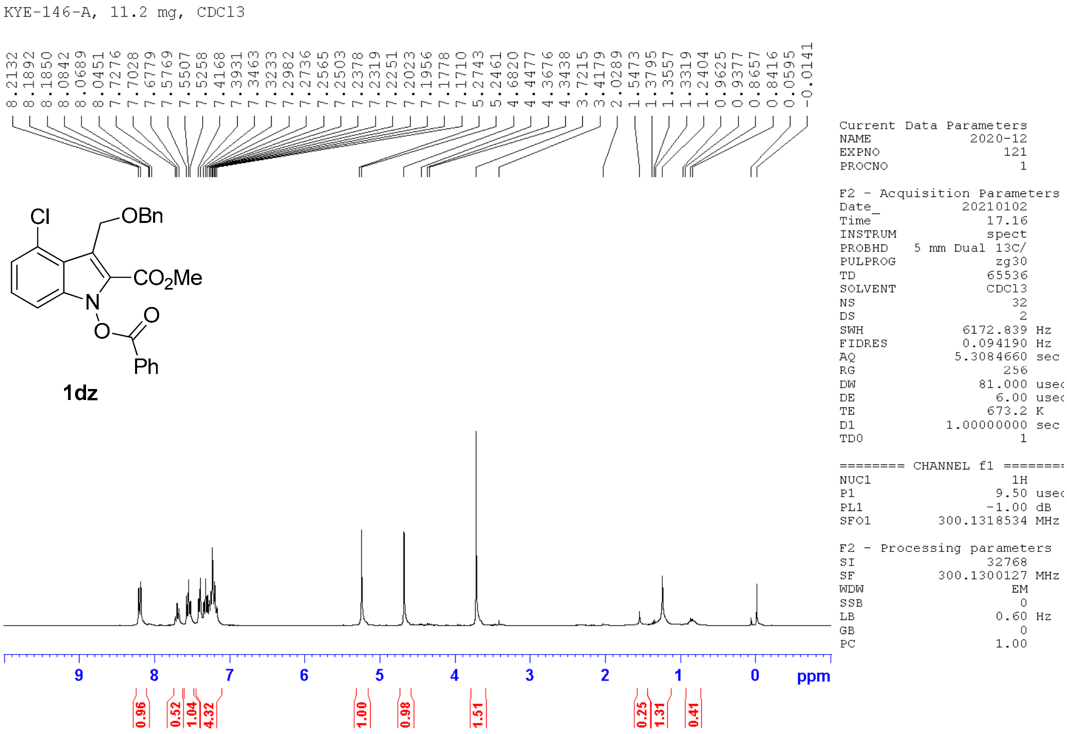

^{1}H NMR spectrum (300 MHz, $CDCl_3$) of compound 1dz

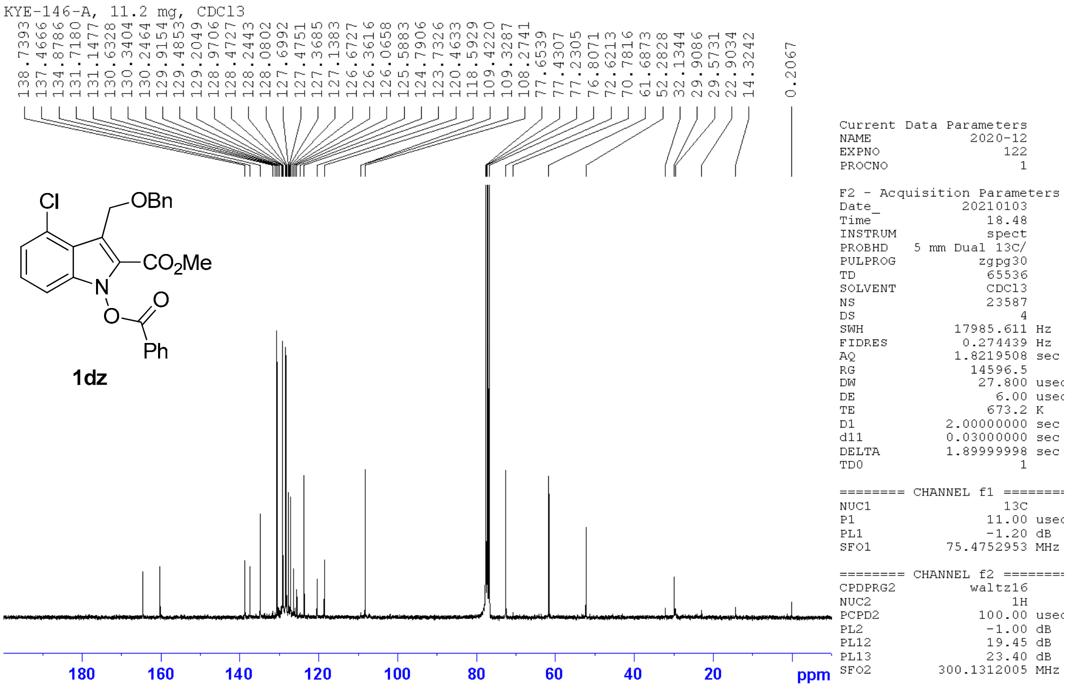

^{13}C NMR spectrum (75 MHz, $CDCl_3$) of compound 1dz

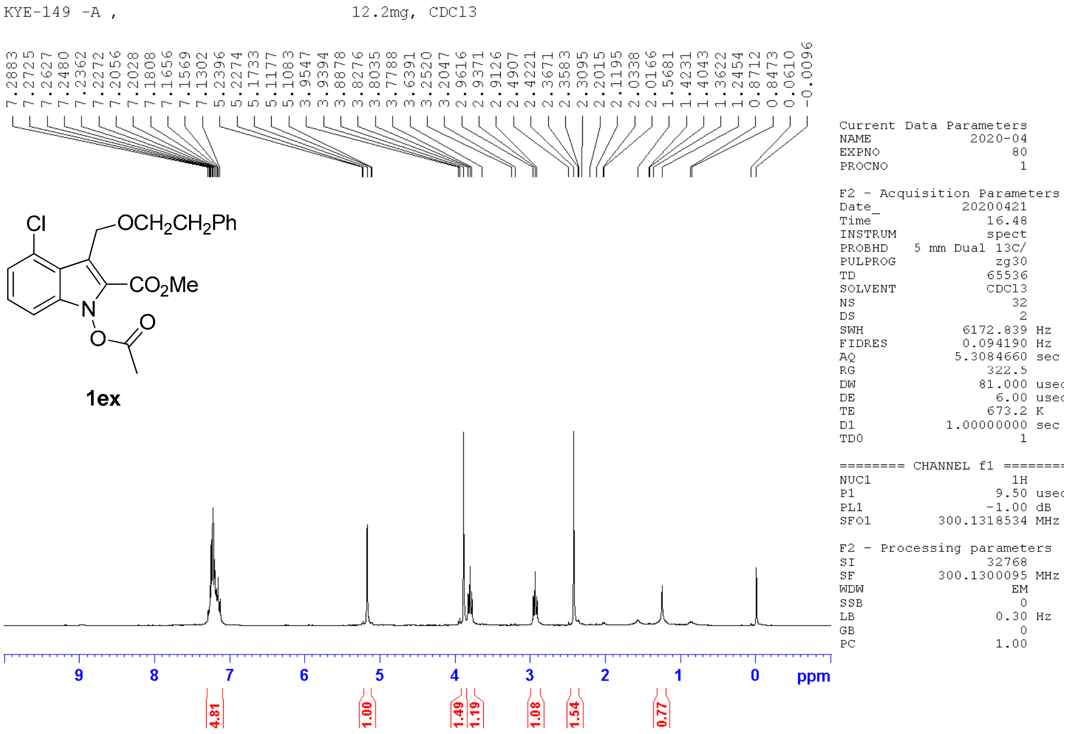

^{1}H NMR spectrum (300 MHz, $CDCl_3$) of compound 1ex

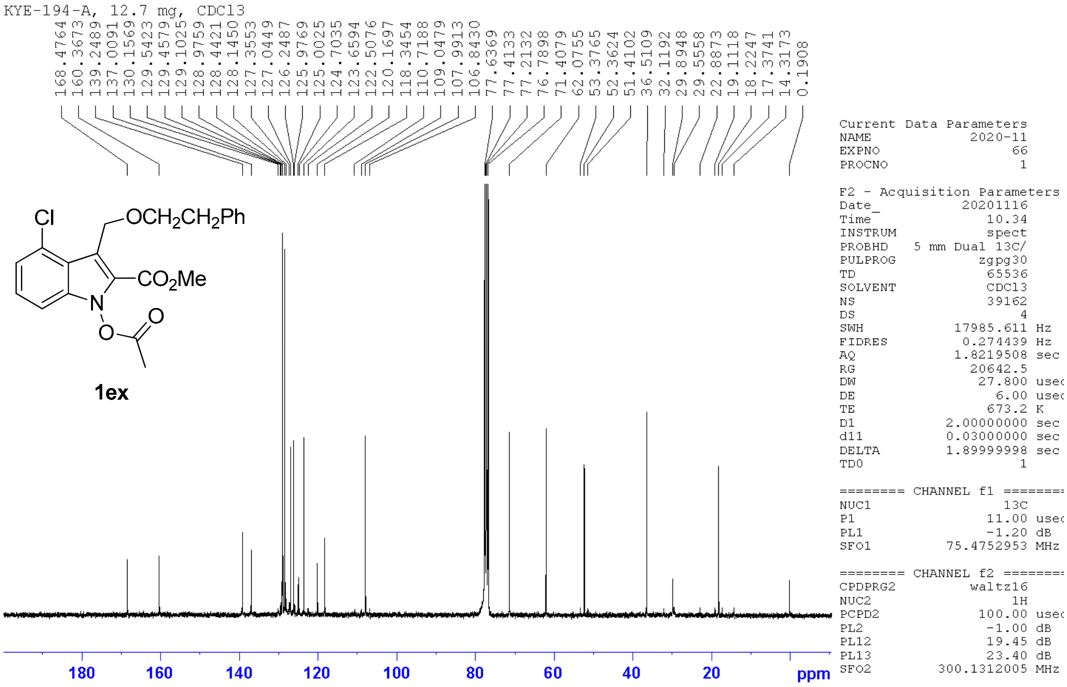

^{13}C NMR spectrum (75 MHz, $CDCl_3$) of compound 1ex

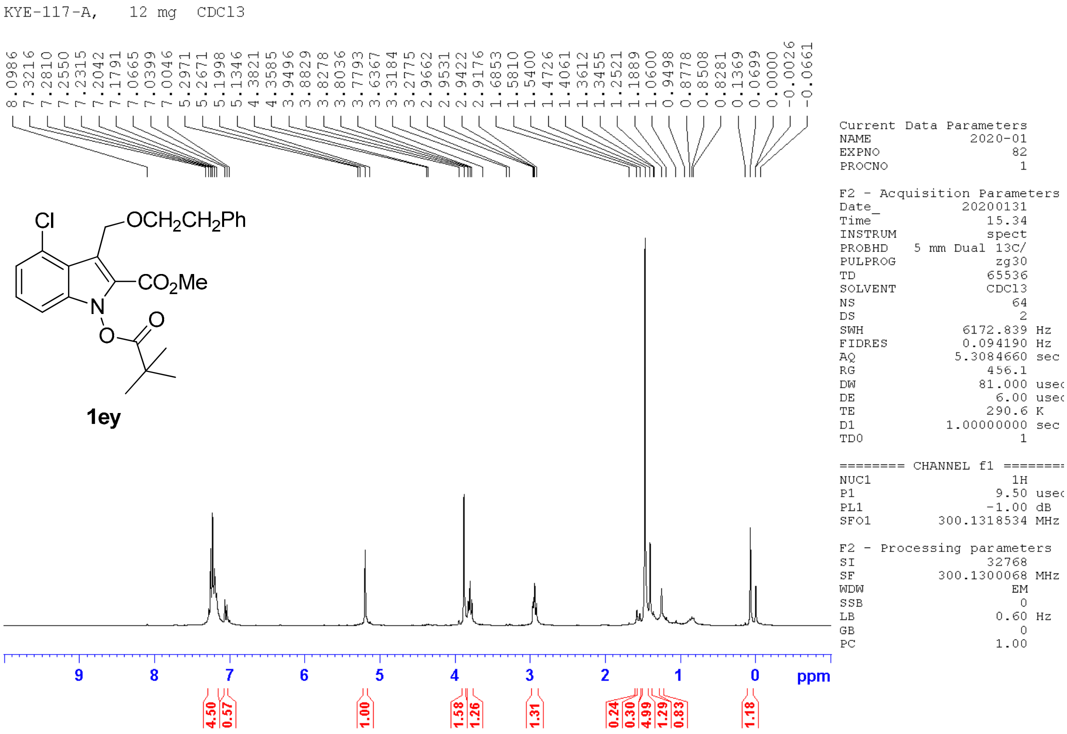

^{1}H NMR spectrum (300 MHz, $CDCl_3$) of compound 1ey

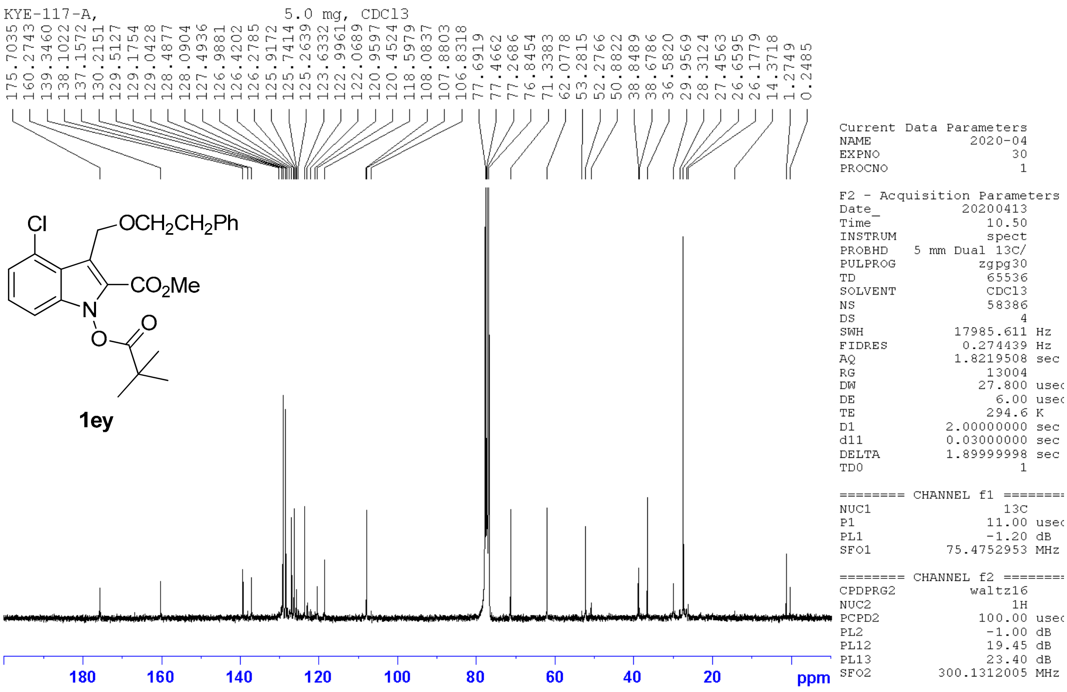

^{13}C NMR spectrum (75 MHz, $CDCl_3$) of compound 1ey

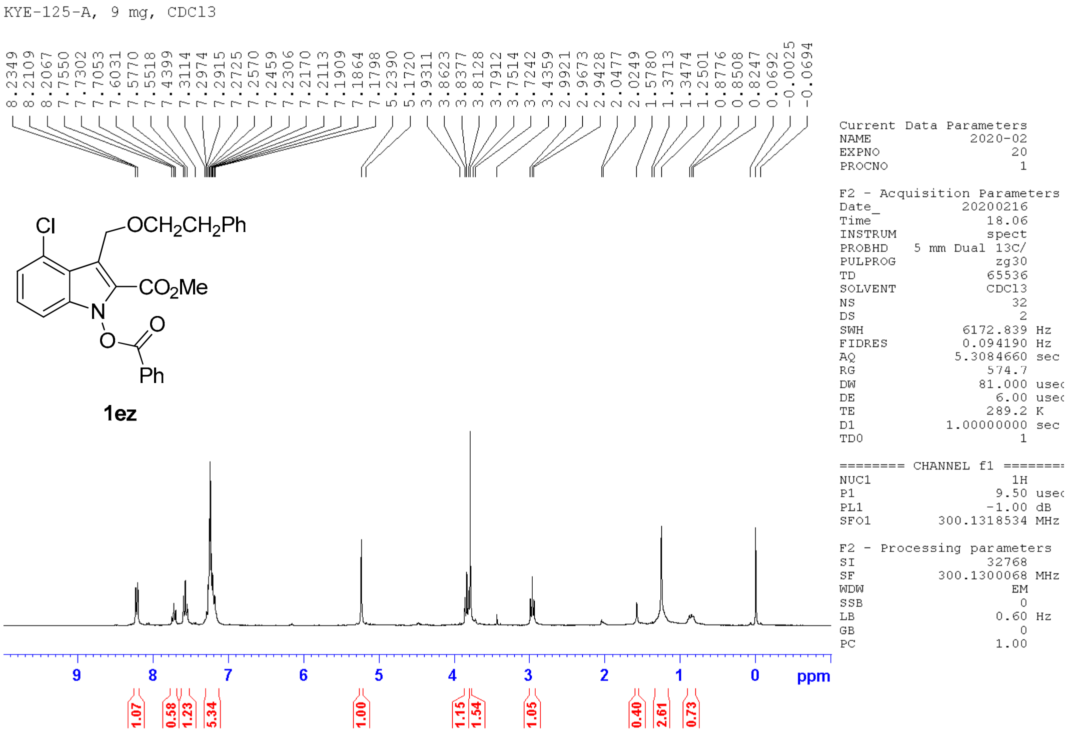

^{1}H NMR spectrum (300 MHz, $CDCl_3$) of compound 1ez

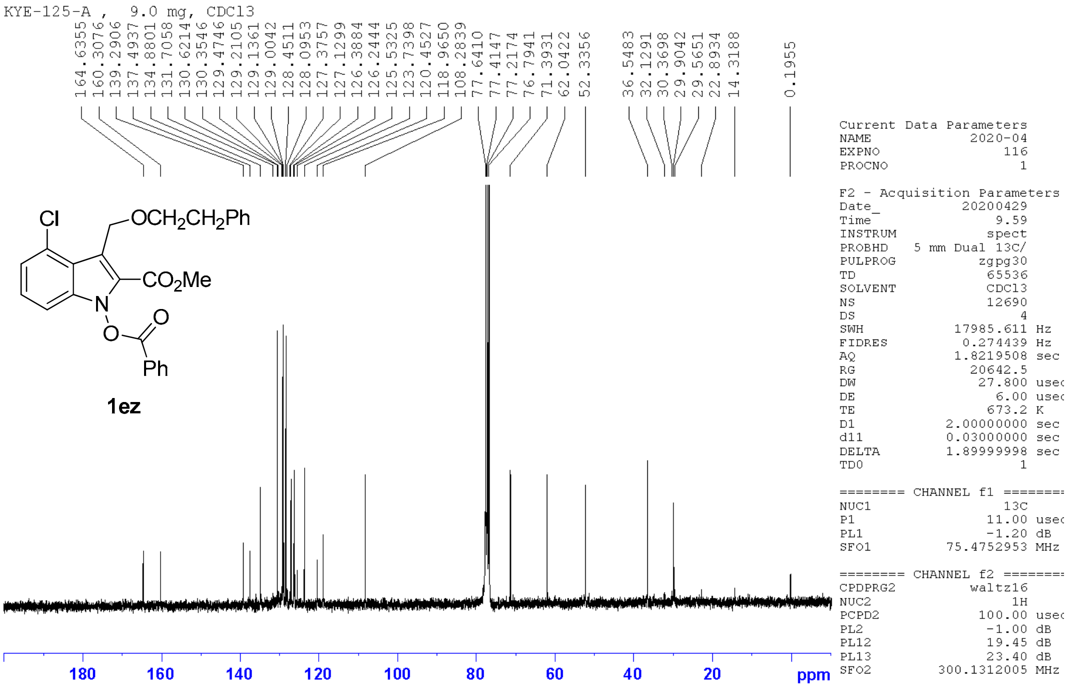

^{13}C NMR spectrum (75 MHz, $CDCl_3$) of compound 1ez

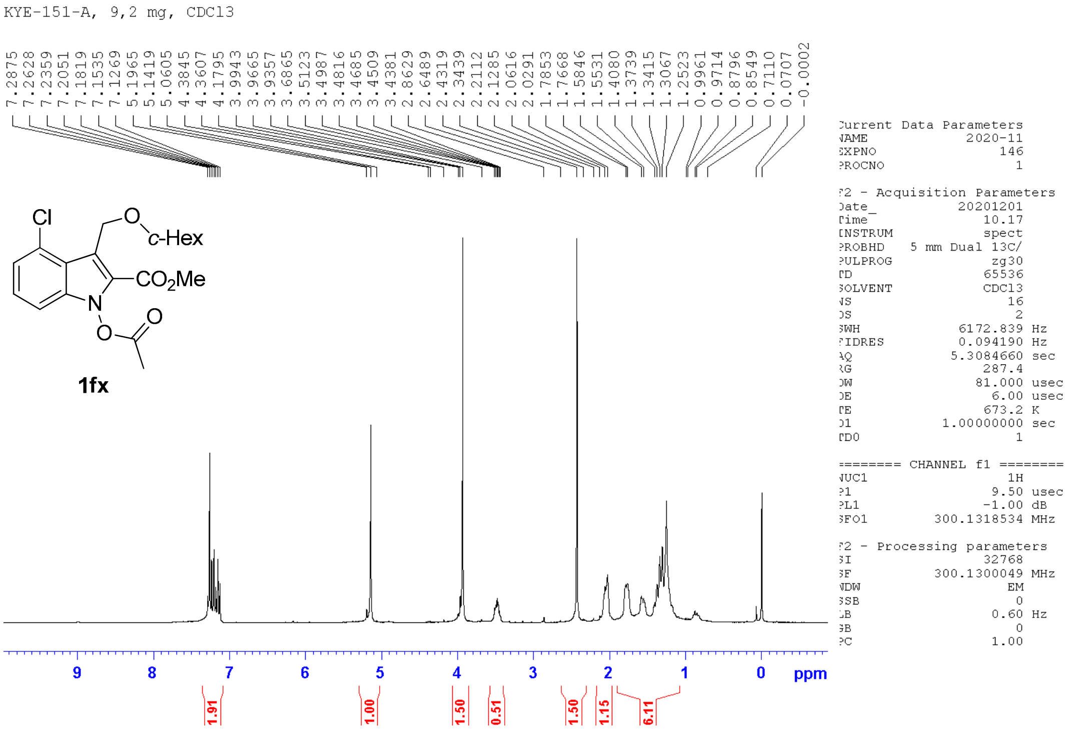

^{1}H NMR spectrum (300 MHz, $CDCl_3$) of compound 1fx

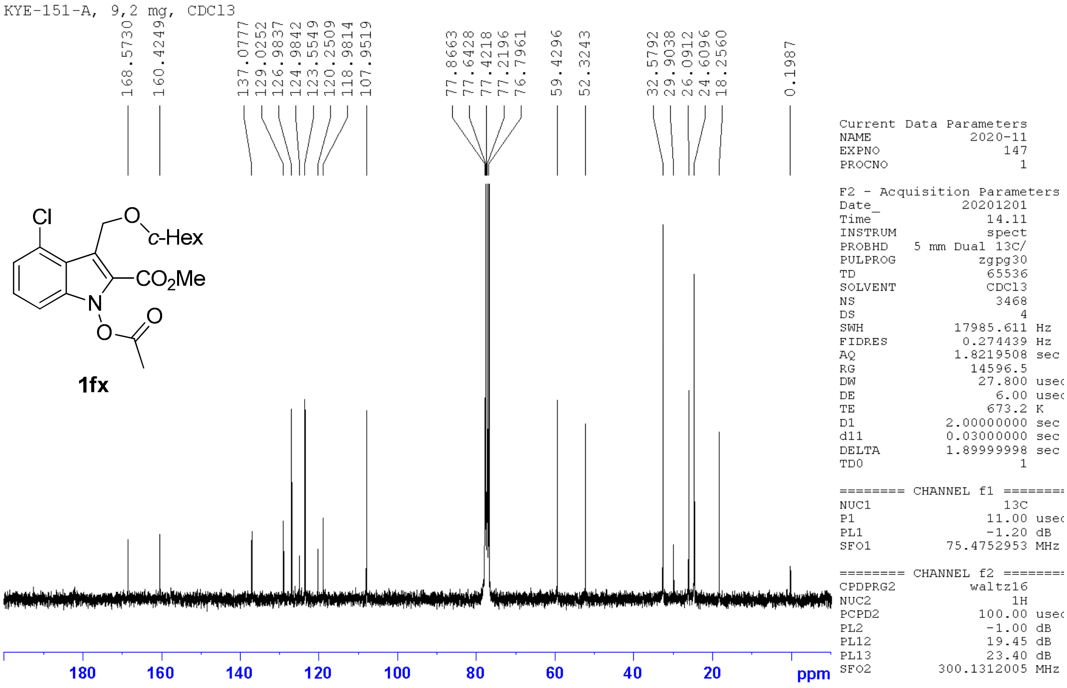

^{13}C NMR spectrum (75 MHz, $CDCl_3$) of compound 1fx

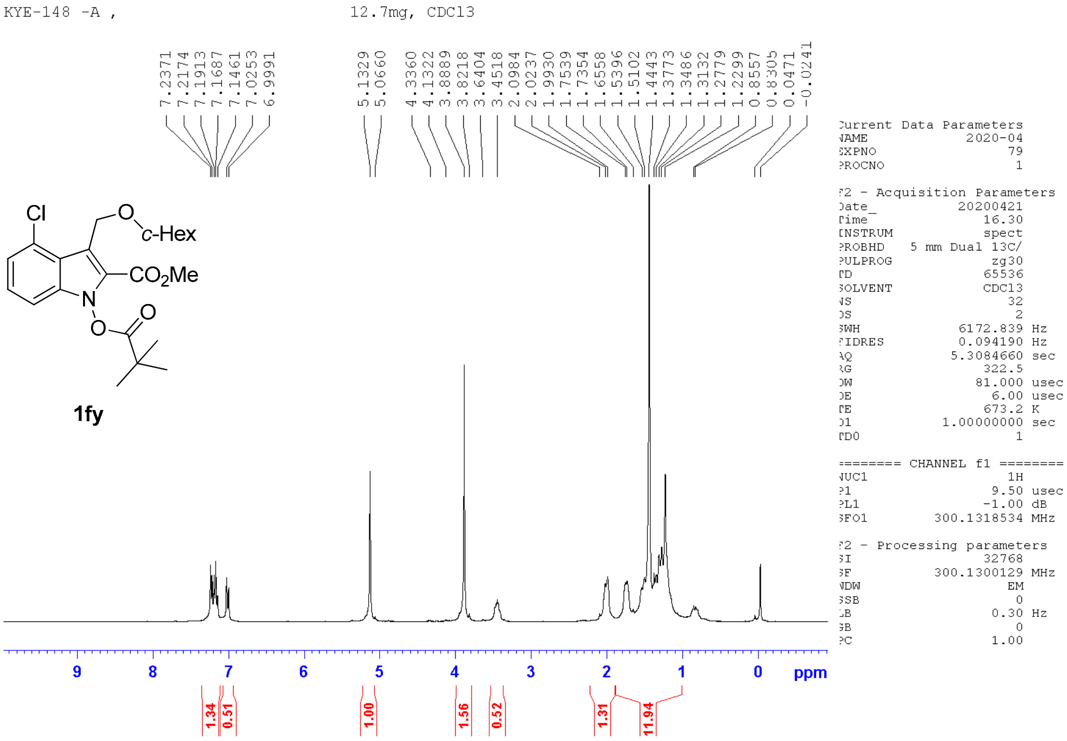

^{1}H NMR spectrum (300 MHz, $CDCl_3$) of compound 1fy

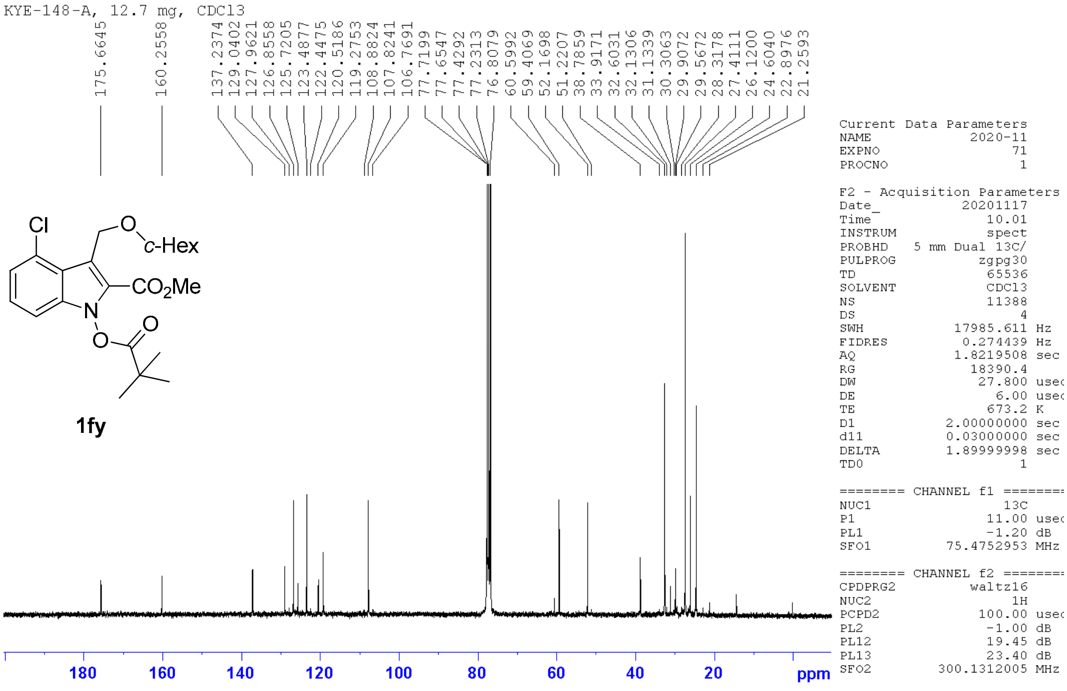

^{13}C NMR spectrum (75 MHz, $CDCl_3$) of compound 1fy

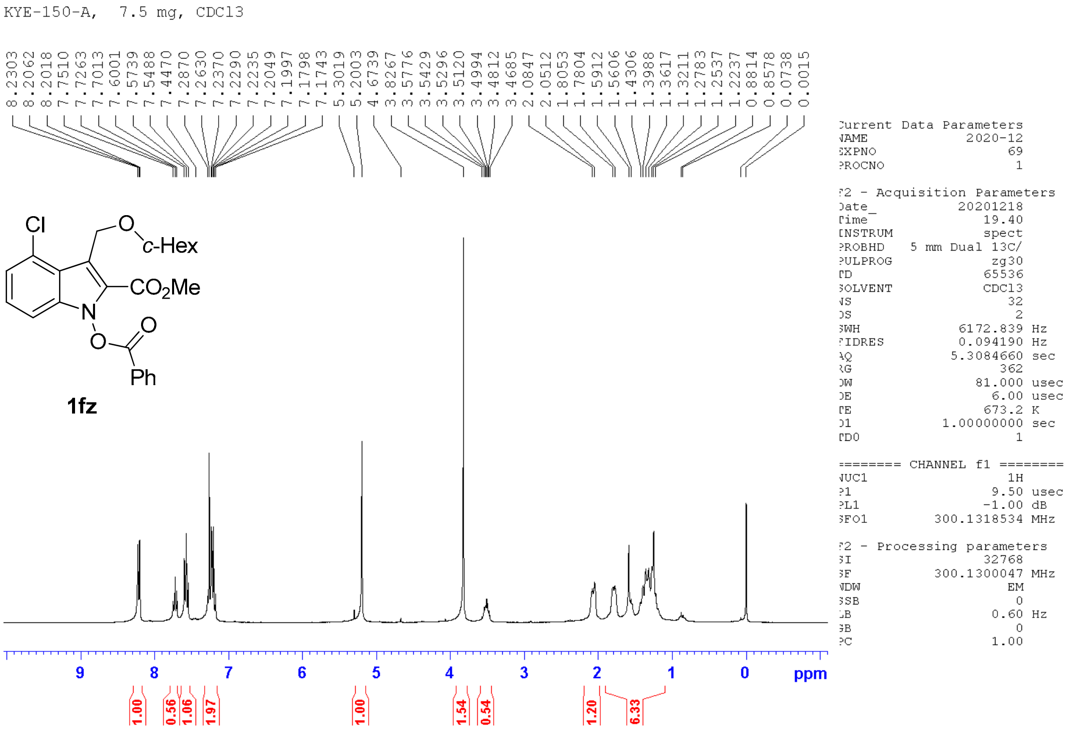

^1H NMR spectrum (300 MHz, $CDCl_3$) of compound 1fz

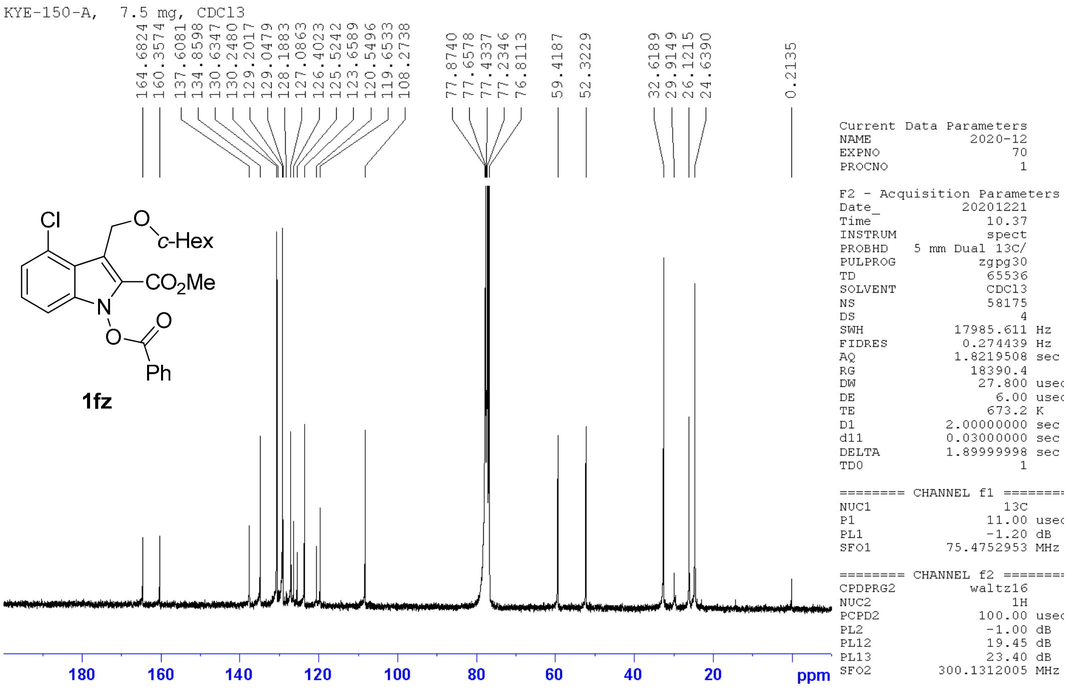

^{13}C NMR spectrum (75 MHz, $CDCl_3$) of compound 1fz
